# Role of Polyamines as Biomarkers in Lymphoma Patients: A Pilot Study

**DOI:** 10.3390/diagnostics12092151

**Published:** 2022-09-04

**Authors:** Donatella Coradduzza, Adriana Ghironi, Emanuela Azara, Nicola Culeddu, Sara Cruciani, Angelo Zinellu, Margherita Maioli, Maria Rosaria De Miglio, Serenella Medici, Claudio Fozza, Ciriaco Carru

**Affiliations:** 1 Department of Biomedical Sciences, University of Sassari, 07100 Sassari, Italy; 2 Department of Clinical and Experimental Medicine, University of Sassari, 07100 Sassari, Italy; 3 Institute of Biomolecular Chemistry, National Research Council, 07040 Sassari, Italy; 4 Department of Medical, Surgical and Experimental Sciences, University of Sassari, 07100 Sassari, Italy; 5 Department of Chemistry and Pharmacy, University of Sassari, 07100 Sassari, Italy; 6 University Hospital of Sassari (AOU), 07100 Sassari, Italy

**Keywords:** biomarkers, polyamines, lymphoma

## Abstract

Lymphomas represent a heterogeneous and widely diversified group of neoplastic diseases rising from a variety of lymphoid subsets at heterogeneous differentiation stages. These lymphoproliferative disorders lead to the clinicopathological complexity of the classification of lymphoid neoplasms, describing to date more than 40 categories of non-Hodgkin’s lymphoma (NHL) and 5 categories of Hodgkin’s lymphoma (HL). Inflammation has been shown to play a key role in the evolution of cancer diseases, and it might be interesting to understand their role also in the context of lymphoid neoplasms. Among circulating biomarkers, the role of polyamines belonging to the arginine and lysine metabolism is relevant. Through modern analytical methods, such as mass spectrometry (MS), we are enabled to increase knowledge and improve our understanding of cancer metabolism. In this study, high-resolution mass spectrometry was used in combination with high-performance liquid chromatography (LC-HRMS) to measure serum levels of polyamines and identify possible diagnostic circulating biomarkers, potentially allowing a more accurate assessment of the diagnostic stratification of lymphoma patients and robust comparisons between different patient groups.

## 1. Introduction

### 1.1. Lymphoma: Symptoms and Causes

Lymphomas represent a heterogeneous and diverse group of neoplastic diseases of the lymphocytes from which they derive [1]. They are classified according to the cell of origin [2]. The diversity of lymphoma subtypes reflects the intricate network of physiological processes that give rise to numerous lymphoid subsets differing in differentiation lines (B, T, and NK) and degree of functional maturation [3]. These lymphoproliferative disorders lead to clinicopathological complexities [4,5]. The current World Health Organization (WHO) classification of lymphoid neoplasms describes more than 40 categories of non-Hodgkin’s lymphoma (NHL) and 5 categories of Hodgkin’s lymphoma (HL) [6]. As of 2016, the classification incorporates information from clinical, morphological, immunophenotypic, molecular, and genetic findings but remains too heterogeneous on disease biology [7]. Non-Hodgkin’s lymphomas are first identified based on the cell of origin (B, T, or NK lymphocytes) and then on morphological, immunophenotypic, genetic, and molecular criteria integrated with clinical features [8]. HL is characterized by the presence of neoplastic cells within a large polymorphic reactive microenvironment (composed of eosinophils, lymphocytes, plasma cells, fibroblasts, and collagen fibers), of which neoplastic cells often represent only a fraction (approximately 1%) [7]. In all cases, the diagnosis of lymphoma should be based on a histological examination of appropriate biopsy material [9]. An incisional or excisional biopsy is always recommended [10]. An integrated diagnostic approach is one that allows a diagnosis, including a morphological examination of peripheral blood smear and bone marrow aspirate, immunophenotypic characterization of circulating lymphocytes by cytofluorimetry, histological and immunohistological evaluation of bone marrow biopsy, by experienced hematopathologists [11]. Among clinical prognostic factors, IPI, International Prognostic Index for Diffuse Large B-cell Lymphoma, remains a useful and reliable clinical tool for measuring the extent and aggressiveness of the disease in lymphoma patients [12]. High IPI scores are associated with a worse prognosis, but this index is not useful in identifying the individual high-risk patient [13]. There is a need to further explore additional factors, including tumor biology and/or the metabolic environment of the host [14].

### 1.2. Diagnosis: Inflammation Biomarkers

New cellular and molecular biomarkers that can be used in clinical decision-making are becoming increasingly important [5,15]. The discovery of biologically relevant biomarkers and therapeutic targets is a fundamental objective of precision medicine [16]. Inflammation has been shown to play a key role in the evolution of cancer diseases, and some authors have studied the role of several biomarkers of inflammation [17], such as neutrophil/lymphocyte ratio (NLR), derived NLR [dNLR = neutrophils/(white blood cells − neutrophils)], platelet/lymphocyte ratio (PLR), monocyte/lymphocyte ratio (MLR), (neutrophil × monocyte)/lymphocyte ratio (SIRI) and (neutrophil × monocyte × platelet)/lymphocyte ratio (AISI). An increasing number of studies have found that a high absolute monocyte count (AMC), decreased absolute lymphocyte count (ALC), absolute lymphocyte to absolute monocyte count (AML), neutrophil to lymphocyte ratio (NLR), platelet to lymphocyte ratio (PLR) and other combined ratios may be predictive and/or prognostic in various solid tumors [18,19,20,21,22]. The AISI value was found to be a predictor of patient outcome compared to other inflammatory indices [21]. Similar results have been demonstrated in diffuse large B-cell lymphoma (DLBCL) [22] and classical Hodgkin’s lymphoma (cHL) [23]. Despite the presence of many hematological prognostic indices, the clinical course and overall survival are often highly variable, even within the same subgroup of patients [24]. Recent studies suggest that simple, low-cost, low-risk tests such as neutrophil-to-lymphocyte ratio (NLR) and lymphocyte-to-monocyte ratio (LMR) can be used to assess prognosis [25]. A combined analysis with metabolites allows fingerprints of biochemical activity in tissues and organs, which correlate directly with cellular phenotypes [26].

### 1.3. Polyamines

In recent years, many authors have focused on the study of metabolic processes in cancer cells. Among the circulating biomarkers, the role of polyamines belonging to the arginine and lysine metabolism is relevant [27,28,29]. Polyamines are essential for normal cell proliferation, gene expression, membrane stabilization, apoptosis, and organogenesis [30,31,32]. Malignant transformation of normal cells requires an increase in cell proliferation; thus, polyamine concentration increases during malignant transformation [33,34,35,36,37]. Modern analytical methods, such as mass spectrometry (MS), have enabled researchers to increase their knowledge and improve their understanding of cancer metabolism [1,2]. In this study, high-resolution mass spectrometry was used in combination with high-performance liquid chromatography (LC-HRMS) to measure serum levels of polyamines [3]. The study aims to identify possible diagnostic circulating biomarkers, allowing a more accurate assessment of the diagnostic stratification of lymphoma patients and robust comparisons between different patient groups to develop an integrated clinical-biological index to ideally improve patient therapeutic management [3].

## 2. Materials and Methods

### 2.1. Clinical Characteristics of Patients

This study examined 10 patients with Hodgkin’s lymphoma and 63 with non-Hodgkin’s lymphoma from the hematology department of the Department of Hematology, University Hospital of Sassari, versus 73 samples of healthy volunteers from the transfusion center of the Health Protection Agency of Sassari. Human serum samples were collected and frozen at −80 °C until use. After incubation of the blood sample at 37 °C for 60 min, the serum was separated by centrifugation at 2000 RPM for 10 min and then stored at −80 °C. Patients enrolled in our study were only diagnosed with de novo forms, excluding those forms developed in immunosuppressed patients (HIV positive or transplanted).

### 2.2. Serum Samples Preparation

The studies were conducted in accordance with the Declaration of Helsinki. Written informed consent was obtained from each subject before the study. Table 1 shows detailed information on demographics and a clinical-pathological patient list of the clinical patient’s characteristics. All serum samples were stored at −80 °C from the collection until measurement. An aliquot of 250 μL of serum was transferred into an Eppendorf microtube and mixed with 150 μL of methanol (containing 0.05% HFBA) and 100 μL of water for 50 s. After precipitation, samples were centrifuged for 9 min at 15,000 rpm and frozen overnight at −20 °C. The supernatant was evaporated to dryness at 36 °C under a stream of nitrogen. The residue was reconstituted into 500 μL of mobile phases and 50 μL of IS (internal standard, deuterated histamine). An aliquot of 5μL of the solution was injected into the LC-HMRS system for analysis. Serum sample preparation was performed in the same manner as the quality control (QC) samples. The supernatants obtained from these solutions were also used as the QC samples. The QC sample is a mixture of all samples, containing all information in the serum samples, and it was used to optimize and supervise the injection process. QC samples were injected occasionally to test the stability of both the samples and the system during acquisition. Prior to sample analysis, the QC samples were injected six times to monitor the stability of the instrument. The six QC samples were then processed in parallel and injected to assess the repeatability of the method.

### 2.3. Statistical Analysis

Results are expressed as an average value (mean ± DS). The distribution of variables was evaluated using the Kruskal–Wallis rank and applied in order to compare the groups. Kruskal–Wallis rank-sum was employed to evaluate the distributions of each variance in the three groups under observation, assuming the value *p* < 0.05 as statistically significant. Statistical comparisons among the groups of parametric variables were evaluated using the unpaired Student *t*-test. The non-parametric continuous variables were compared with the case of normally distributed samples and with the median ± median absolute deviation (MAD) in the case of non-normal sample distribution. Correlations among variables were estimated using a Pearson correlation. A supervised analysis was carried out by applying the orthogonal partial discriminant analysis of the minimum square (OPLS-DA), representing a rotation of the corresponding PLS-DA models and simplifying the information into only one predictive component, while maintaining the same predictive capacity [4]. To avoid model overfitting, the corresponding PLS-DA models were validated by 300 permutation tests [5]. The prediction strength of the model was evaluated by the “Leave out” analysis [6]. Variable importance parameter (VIP) values were used to assess the overall contribution of each X variable to the model, summed over all components and weighted according to the Y variation, accounted for by each component [7]. The number of terms in the sum depends on the number of PLS-DA components found to be significant in distinguishing the classes. The Y axis indicates the VIP scores corresponding to each variable on the X axis [7]. Bars indicate the factors with the highest VIP scores and thus are the most contributory variables in class discrimination in the PLS-DA model. The statistical analysis was carried out using Stagraphics Centurion XVII (v.17.2) and SIMCA-P version 14.0 (Umetrics AB, Umea, Sweden).

## 3. Results

### 3.1. Patients and Data

**Figure 1:**
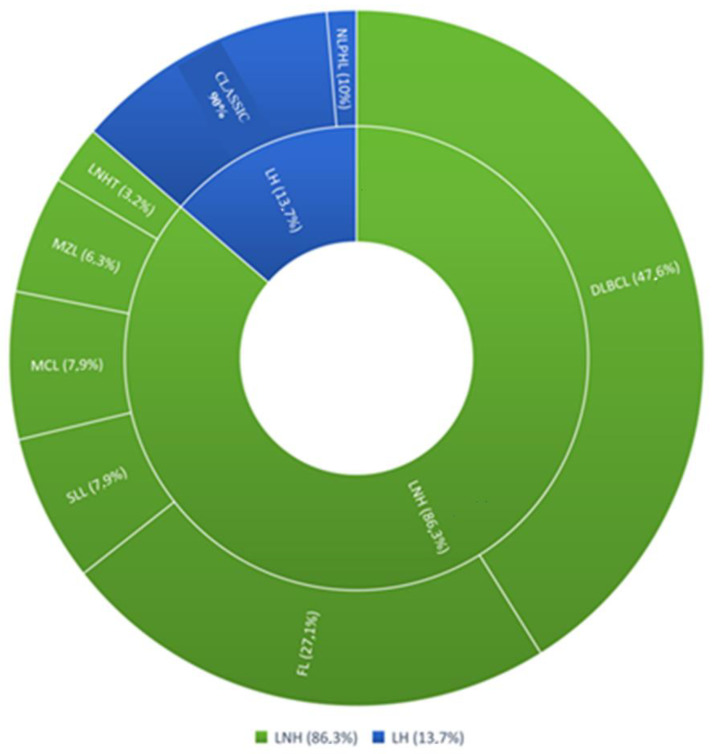
Clinical case study. NHL (non-Hodgkin’s lymphoma); subtypes: DLBCL (diffuse large B cell lymphoma), FL (follicular lymphoma), SLL (small lymphocytic lymphoma), MCL (mantle cell lymphoma), MZL (marginal zone lymphoma), LNH T (non-Hodgkin T cell lymphoma). HL (Hodgkin lymphoma); subtypes: CHL (classical Hodgkin lymphoma), NLPHL (nodular lymphocyte predominant Hodgkin lymphoma).

The mean age of patients was 59.04 years (±14.61); 38 (52%) were male, and 35 (47%) were female. In total, 63 patients (86.3%) had a diagnosis of non-Hodgkin’s lymphoma, and 10 patients (13.7%) had a diagnosis of Hodgkin’s lymphoma. Among NHL subjects, 30 patients (41.2%) had diffuse large B-cell lymphoma (DLBCL), 17 patients (23.3%) had follicular lymphoma (FL), 5 patients (6.8%) had small lymphocyte lymphoma (SLL), 5 patients had mantle cell lymphoma (MCL), 4 patients had marginal zone lymphoma (MZL), 2 patients (2.7%) had T-cell non-Hodgkin’s lymphoma. Among Hodgkin’s lymphomas, 9 patients (12.3%) represented the classic variant (CHL), and only 1 patient presented with the nodular lymphocyte-predominant variant (NLPHL) (Figure 1).

**Figure 2:**
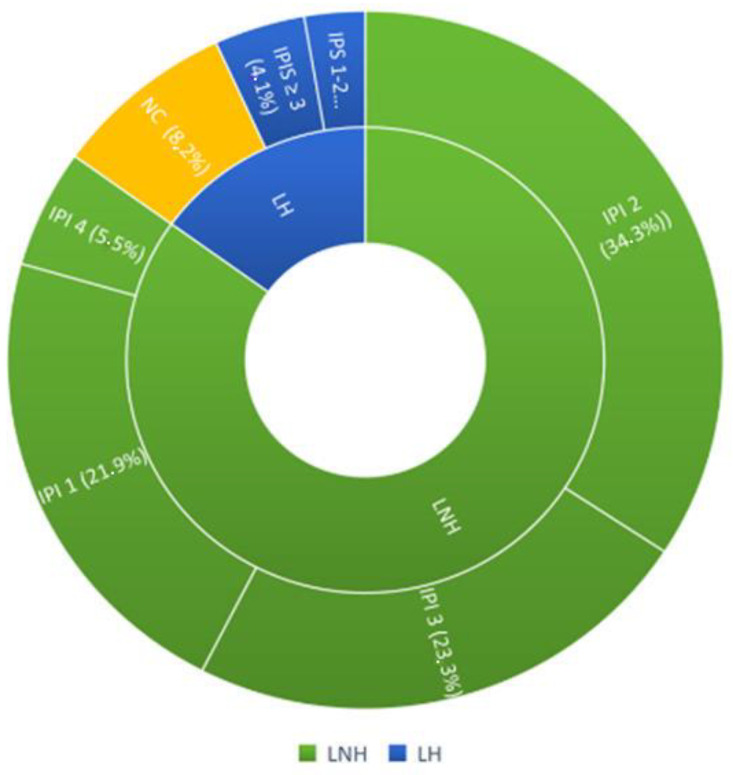
Representation of the prognostic stratification of the patients included in the study.

The patients, according to Ann Arbor staging, were divided into 9.58% (7 pcs) stage I; 19.17% (14 pcs) stage II; 23.28% (17 pcs) stage III; 47.94% (35 pcs) stage IV. In addition, patients were stratified according to their prognostic risk using the IPI (International Prognostic Index) score for non-Hodgkin’s lymphoma and the IPS-Hasen clever index score for Hodgkin’s lymphoma. For non-Hodgkin’s lymphomas, the breakdown was as follows: 16 patients (21.9%) had an IPS 1, 25 patients (34.3%) had an IPS 2, 17 patients (23.3%) had an IPS 3, 4 patients (5.5%) had an IPS 4. Among Hodgkin’s lymphomas, 2 patients (2.7%) had an IPS 1-2 score, 3 patients (4.1%) had an IPS ≥ 3. Six HL patients were excluded from the prognostic classification because they had stages of non-advanced disease (Figure 2).

### 3.2. Biochemical Parameters

Table 1 shows the clinical, functional and biochemical parameters: age, sex, blood cell count (WBC, RBC, HGB, PLT), white blood cell count (lymphocytes, neutrophils, monocytes), plasma inflammatory indexes (LMR, NLR, PLR) and combined plasma inflammatory indices (SIRI, AISI). We found a significant difference between patients of the three groups (LNH, HL and controls) for all values of the hemochrome-cytometric examination (WBC, RDW, PLT absolute count of neutrophils, lymphocytes, and monocytes) except hemoglobin and for all parameters of inflammation (MRL, NLR, PLR, SIRI, AISI) (Table 1 and Supplementary Figure S1). The value of WBC, neutrophil platelets, monocytes, lymphocytes was increased in lymphoma patients (LNH and HL) compared to healthy patients. Among the parameters of inflammation, LMR, NLR, PLR, SIRI, and AISI were increased in lymphoma patients (LNH and HL) compared to healthy patients, where the combined plasma inflammatory indices (SIRI, AISI) were particularly high. The AISI value in lymphoma patients reached values > 3000. Table 2 shows the clinical parameters of lymphoma patients (LNH and HL), according to Ann Arbor classification, presence of B symptoms, bone marrow infiltration, HBV and HCV infection. No parameter showed a statistically significant difference between the two groups, thus confirming a high degree of homogeneity between patients with NHL and HL.

### 3.3. Serum Levels of Polyamines, Related Amino Acids and Metabolites

Table 3 shows the levels of plasma polyamines, related amino acids (arginine, lysine) and metabolites (GABA) in the three groups (NHL, HL and HEALTHY). There are significant differences between the three groups for the following parameters: putrescine, spermidine, acetyl-putrescine, acetyl-spermidine, acetyl-spermine, ornithine, s-adenosyl-methionine, and GABA.

Putrescine, spermidine, acetyl-putrescine, acetyl-spermidine, ornithine, and GABA were found to be increased in lymphoma patients (NHL and HL) compared to healthy patients, whereas acetyl-spermidine and s-adenosyl-methionine were found to be increased in healthy patients compared to lymphoma patients, NHL, and HL (Supplementary Figure S2). We evaluated statistically significant polyamines between the three observation groups when comparing NHL and HL using Student’s unpaired *t*-test and found a significant difference for acetyl-spermine (*p*-value 0.008), which was increased in NHL patients compared to HL patients. The box plots of comparison, shown in Supplementary Figure S3, between NHL and HL are reported for the polyamines found to be statistically significant using the Kruskal–Wallis test: acetyl-putrescine, acetyl spermidine, acetyl-spermine, putrescine, spermidine, ornithine, S-adenosyl methionine and GABA.

### 3.4. Multivariate Analysis

**Figure 3:**
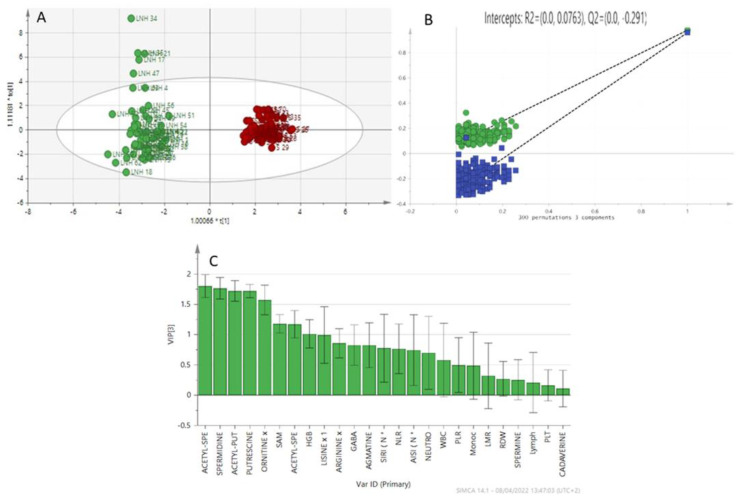
(**A**) OPLS-DA model patients with NHL (green) and healthy patients (red). (**B**) The variable importance in projection (VIP) highlights the differences between the two groups (LNH vs. HEALTHY). (**C**) Validation model.

**Figure 4:**
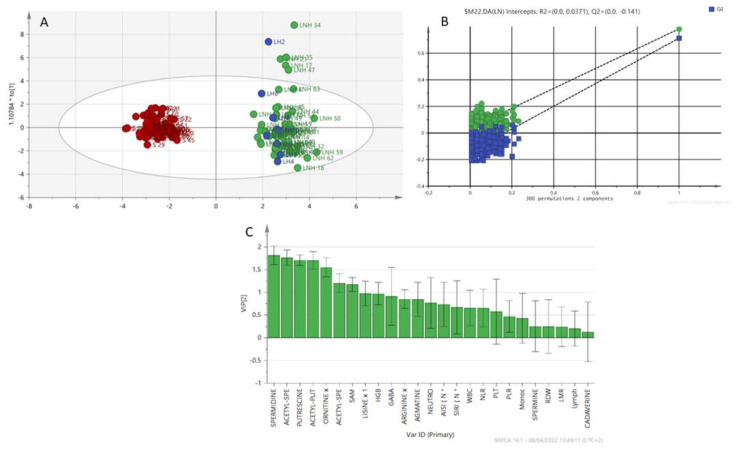
(**A**) OPLS-DA, NHL patients (green), LH patients (blue) and healthy patients (red). (**B**) Variable importance in projection (VIP) plot: important features identified by OPLS-DA in descending order of importance. The graph represents the relative contribution of parameters to the variance between individuals. High value of VIP score indicates great contribution to the group separation (LNH+LH vs. healthy). (**C**) Permutation (LNH+LH vs. healthy).

In order to better understand the role of polyamines in lymphoma patients, we performed a multivariate analysis by analyzing the data obtained from the patient’s serum integrated with the patient’s clinical data. In Figure 3A, the multivariate analysis method, using the OPLS-DA (Orthogonal partial least squares discriminant analysis), shows good discrimination between the two groups: non-Hodgkin’s lymphoma samples and healthy subjects; it shows how the two groups naturally form two clusters. The supervised analysis was performed by applying orthogonal least square partial discriminant analysis (OPLS-DA), which implies a rotation of the corresponding PLS-DA models and simplifies the information into a predictive component, while maintaining the same predictive capacity. Variable Importance in Projection (VIP), obtained from the OPLS-DA model, highlights the contribution of the analytes, Figure 3C, in discriminating groups in the OPLS-DA model. The *Y*-axis shows the VIP scores corresponding to each variable on the *X*-axis, using VIP > 1 as the selected parameters. Among the polyamines, acetyl-spermidine, spermidine, acetyl-putrescine, putrescine, and ornithine are the variables that discriminate these two groups the most. The analysis is in agreement with the results obtained with the Kruskal–Wallis test for acetyl spermidine, spermidine, acetyl-putrescine, putrescine and ornithine (*p* < 0.05). To avoid model overfitting, the OPLS-DA models were validated with a 300-fold permutation, Figure 4B. The resulting regression lines showed an R2 intercept at 0.0763 and a Q2 intercept at −0.291, indicating a valid model.

The analysis was also extended to patients with Hodgkin’s lymphoma. The group of lymphoma patients (LNH and LH) was compared with the group of healthy patients (Figure 4). The model shows a natural separation between healthy subjects and subjects with lymphomas (LNH + LH). The VIP scoring variables are shown in Figure 4C. Acetyl-spermidine, acetyl-putrescine, putrescine, spermidine, and ornithine are the discriminating variables for the groups (LNH+LH vs. Healthy). A permutation testing is shown in Figure 4B to validate the classification model. The resulting regression lines showed an R2 intercept at 0.0371 and a Q2 intercept at −0.141.

Outliers can be seen in the above projections (Figure 3 and Figure 4). We analyzed the clinical characteristics of outlier LNH 34, a patient with a particularly aggressive LNH subtype (DLBCL anaplastic variant CD30+) associated with a particularly high AISI (3450.20) and an NLR of 9.27. The patient also had a previous HCV infection. Similarly, outlier LNH 35 and LNH 21 had the same subtype as LNH 34 (DLBCL) with particularly high AISI (2105.79 and 1694.81 respectively), NLR of 4.31 and 9.84, respectively, and previous HCV infection. LNH 17 and LNH 47 were diagnosed with SLL and MCL, respectively, stage IV disease onset and bone marrow infiltration. LNH 17 presented with AISI 1074.34 and an NLR of 7.79. LNH 47 presented at diagnosis with lymphoid hyperleukocytosis (WBC 42.500/mmc, L 33.400/mmc) and extremely low AISI and NLR of 39.23 and 0.07, respectively. The LH 2 outlier is a patient with a classic variant of Hodgkin’s lymphoma, clinically aggressive (stage IV, symptoms B, IPS 4) neutrophil hyperleukocytosis (WBC 26.770/mmc, Neutrophils 19,110/mmc), increased monocyte count (1300/mmc), associated with a fairly high AISI value (1612).

### 3.5. Analysis of LNH Patients with HCV+ Infection

Following the identification of LNH outliers (LNH 34, LNH 35, LNH 21), we wanted to compare HCV+ (positive) and HCV− (negative) non-Hodgkin’s lymphoma patients; the biochemical parameters are shown in Table 4, and the levels of polyamines, related amino acids and metabolites are shown in Table 5. The two groups differed in absolute neutrophil count and MRL value, which were increased in HCV+ patients compared to HCV− patients (Table 4). Acetyl-putrescine differed between HCV+ patients compared to HCV− patients in a statistically significant manner (* *p*-value = 0.008) (Table 5, Supplementary Figure S4).

## 4. Discussion

Lymphomas represent one of the most heterogeneous groups of all malignant tumors in medicine. This heterogeneity is currently a double-edged sword. On the one hand, it has created an evolutionary way of classifying these diseases, and on the other hand, it has allowed the identification of unique biological features. These features have led to new targets and generated an exuberance for new drugs. Heterogeneity has created new subtypes, an ever-increasing number. Each subtype is reclassified and recategorized on the basis of a set of cellular and molecular descriptors. This paradigm has led to an increasingly dense search for new, increasingly personalized diagnostic biomarkers. The study under consideration is based on the search, in amino acid catabolism, for a peculiar biological signature, a sort of fingerprint of this multifaceted pathology. Various diseases, particularly oncological ones, have been associated with increased concentrations of polyamines, with these being considered biomarkers. This study estimated the role of polyamines in the diagnostic and potentially prognostic assessment of lymphomas. Using liquid chromatography combined with high-resolution mass spectrometry (LC-HRMS), we were able to identify some polyamines that appear to differ between lymphoma patients and healthy controls. The multivariate analysis investigated how these two populations (healthy and lymphoma) tend to cluster naturally. The results indicate that the profile of analytes in serum could provide information not only on general metabolic changes but also on clinical outcomes in lymphoma. Studying the individual analytes in a differential global profile, we found that the concentrations of both the acetylated polyamines, acetyl-spermidine and acetyl-putrescine, and the non-acetylated polyamines, spermidine, putrescine and ornithine were significantly different between healthy and lymphoma patients. Comparing NHL and LH patients, we found that spermidine differed between the two groups, although the small number of LH patients resulted in an unbalanced data set. The results show that inflammation parameters, such as NLR, PLR, LMR, SIRI and AISI, can be independently considered valid circulating diagnostic markers in lymphoma. They are easily included in the initial evaluation of the hematological patient. In addition, outlier results suggest that a certain polyamine pattern may reflect a more aggressive disease and also provide potential information on various host factors. Our study is not without limitations due to the fact that the samples were analyzed retrospectively and that the group represented by Hodgkin’s lymphomas was numerically too small compared to non-Hodgkin’s lymphomas. Although the results will need to be validated in a larger cohort of patients, the method seems promising and could become a tool to support diagnostic stratification. Furthermore, by extending the study, examining serum samples from patients at various stages of treatment and/or after disease progression or relapse, polyamines could also be useful as prognostic biomarkers, becoming a tool to support prognostic stratification of lymphoma patients, predict the clinical course, discriminate very high-risk patients, and select the best treatment options and personalized therapy. The hope is that behind these outliers, hitherto considered anecdotal in the context of oncology studies, lies the key to a new avenue in the search for new biomarkers. The results suggest that a single biomarker probably could not reflect the heterogeneous picture of lymphomas. A reasonable conclusion could be to include a broader set of biomarkers to build a predictive model in accordance with personalized medicine.

## Figures and Tables

**Table 1:** Clinical parameters between the three groups under observation (NHL, HL and Sani). Kruskal–Wallis test, *p* < 0.05 as statistically significant, indicated by *.

	NHL	HL	HEALTHY	SIGNIFICANCE
**NUM**	63	10	73	
**SEX**	31 F/32 M	4 F/6 M	27 F/46 M	*p*-value = 0.35
**AGE**	61.71 ± 11.98	42.2 ± 18.85	53.65 ± 8.16	*p*-value = 1.91
**WBC**	10.03 ± 10.39	9.37 ± 6.52	5.99 ± 1.48	* *p*-value = 0.002
**HGB**	12.39 ± 1.86	12.87 ± 1.91	14.43 ± 1.01	*p*-value = 5.41
**RDW**	14.75 ± 2.62	13.92 ± 1.67	15.06 ± 0.76	* *p*-value = 0.008
**PLT**	235.62 ± 108.29	295.60± 114.16	217.72 ± 46.62	* *p*-value = 0.043
**NEUT**	5.66 ± 4.52	5.72 ± 5.06	3.51 ± 1.08	* *p*-value = 0.005
**LYMPH**	3.45 ± 9.03	2.61 ± 1.26	2.09 ± 0.80	* *p*-value = 0.0005
**MONO**	0.69 ± 0.86	0.55 ± 0.42	0.37 ± 0.13	* *p*-value = 0.005
**LMR**	9.42 ± 22.91	6.6 ± 6.05	5.78 ± 3.09	* *p*-value = 0.0004
**NLR**	4.52 ± 5.68	2.51 ± 1.84	1.80 ± 0.66	* *p*-value = 0.00002
**PLR**	169.34 ± 132.96	154.8 ± 120.97	112.10 ± 36.28	* *p*-value = 0.019
**SIRI**	3.77 ± 8.76	1.46 ± 1.67	0.69 ± 0.40	* *p*-value = 0.00007
**AISI**	1152.83 ± 3581.13	455.63 ± 498.18	151.20 ± 93.77	* *p*-value = 0.001

**Table 2:** Clinical parameters between the two groups of lymphoma patients (NHL and HL). Student’s *t*-test was applied unpaired between NHL vs. HL, *p* < 0.05 statistically significant.

	NHL	HL	SIGNIFICANCE
**NUM**	63	10	
**STAGE I**	0.09 ± 0.29	0.1 ± 0.31	*p*-value = 0.96
**STAGE II**	0.15 ± 0.36	0.4 ± 0.51	*p*-value = 0.07
**STAGE III**	0.25 ± 0.43	0.1 ± 0.31	*p*-value = 0.29
**STAGE IV**	0.49 ± 0.50	0.4 ± 0.51	*p*-value = 0.59
**CNS involvement**	0.26 ± 0.44	0.2 ± 0.42	*p*-value = 0.64
**HBV**	0.20 ± 0.40	0.1 ± 0.31	*p*-value = 0.43
**HCV**	0.063 ± 0.24	0	*p*-value = 0.41
**SYMPTOMS B**	0.34 ± 0.48	0.5 ± 0.52	*p*-value = 0.36

**Table 3:** Analysis of polyamine levels in the three groups (LNH, LH and HEALTHY). Kruskal–Wallis test significant for *p* < 0.05 indicated with *. ** indicates the *p*-value obtained with Student’s *t*-test unpaired between LNH vs. LH, *p* < 0.05 statistically significant.

	NHL	HL	HEALTHY	SIGNIFICANCE
**POLYAMINES**	63	10	73	
**PUTRESCINE**	13.90 ± 1.27	13.24 ± 1.31	6.69 ± 1.39	* *p*-value < 0.05 ** *p*-value = 0.13
**SPERMIDINE**	9.18 ± 1.83	5.83 ± 0.88	1.03 ± 0.26	* *p*-value < 0.05 ** *p*-value = 5.95
**SPERMINE**	6.04 ± 1.40	6.09 ± 1.37	6.48 ± 2.15	*p*-value = 0.55
**ACETYL-PUTRESCINE**	1.94 ± 0.48	1.85 ± 0.34	0.14 ± 0.05	* *p*-value < 0.05 ** *p*-value = 0.59
**ACETYL-SPERMIDINE**	2.97 ± 0.45	3.06 ± 0.36	0.16 ± 0.12	* *p*-value < 0.05 ** *p*-value = 0.55
**ACETYL-SPERMINE**	1.72 ± 0.38	1.38 ± 0.31	2.56 ± 0.59	* *p*-value < 0.05 ** *p*-value = 0.008
**AGMATINE**	58.98 ± 7.39	57.68 ± 4.25	70.51 ± 14.17	*p*-value = 3.26
**CADAVERINE**	2.35 ± 0.43	2.13 ± 0.52	2.29 ± 0.66	*p*-value = 0.26
**ORNITHINE**	1.93 ± 0.48	1.89 ± 0.42	0.76 ± 0.13	* *p*-value < 0.05 ** *p*-value = 0.79
**LYSINE**	6.11 ± 0.77	6.22 ± 0.84	7.03 ± 0.63	*p*-value = 1.30
**ARGININE**	7.26 ± 0.47	7.26 ± 0.44	6.41 ± 0.98	*p*-value = 1.32
**S-ADENOSYLMETHIONINE**	213.27 ± 35.42	210.26 ± 36.29	339.35 ± 95.88	* *p*-value < 0.05 ** *p*-value = 0.8
**GABA**	39.89 ± 13.43	46.19 ± 13.05	30.69 ± 2.23	* *p*-value < 0.05 ** *p*-value = 0.17

**Table 4:** Clinical parameters comparison of NHL patients (HCV+ and HCV−). Unpaired Student’s *t*-test between HCV+ and HCV− was applied, * *p*-value < 0.05 as statistically significant.

	LNH HCV+	LNH HCV−	SIGNIFICANCE
**NUM**	**4**	**59**	
**WBC**	13.62 ± 6.23	9.79 ± 10.61	*p*-value = 0.48
**HGB**	12.25 ± 2.34	12.40 ± 1.85	*p*-value = 0.87
**RDW**	14.45 ± 2.20	14.81 ± 2.65	*p*-value = 0.78
**PLT**	311.25 ± 108.923	230.49 ± 107.24	*p*-value = 0.15
**NEUT**	10.72 ± 5.15	5.32 ± 4.31	* *p*-value = 0.01
**LYMPH**	1.85 ± 0.90	3.56 ± 9.32	*p*-value = 0.71
**MONO**	0.91 ± 0.77	0.67 ± 0.87	*p*-value = 0.59
**LMR**	38.25 ± 14.60	7.47 ± 74.5	* *p*-value = 0.008
**NLR**	6.37 ± 3.79	4.39 ± 5.79	*p*-value = 0.50
**PLR**	191.5 ± 93.12	167.84 ± 135.70	*p*-value = 0.73
**SIRI**	6.24 ± 4.16	3.60 ± 8.98	*p*-value = 0.56
**AISI**	1814.56 ± 1418.94	1107.97 ± 3684.1	*p*-value = 0.70

**Table 5:** Levels of polyamines, related amino acids and metabolites in LNH patients (HCV+ and HCV−). Student’s *t*-test was applied unpaired between HCV+ and HCV−, * *p* < 0.05 as statistically significant.

	LNH HCV+	LNH HCV−	SIGNIFICANCE
**POLIAMMINE**	**4**	**59**	
**PUTRESCINE**	14.29 ± 1.24	13.88 ± 1.28	*p*-value = 0.53
**SPERMIDINE**	9.82 ± 1.93	9.14 ± 1.84	*p*-value = 0.47
**SPERMINE**	5.07 ± 1.29	6.11 ± 1.39	*p*-value = 0.15
**ACETYL-PUTRESCINE**	2.42 ± 0.18	1.91 ± 0.48	* *p*-value = 0.039
**ACETYL-SPERMIDINE**	3.11 ± 0.40	2.96 ± 0.46	*p*-value = 0.53
**ACETYL-SPERMINE**	1.40 ± 0.34	1.74 ± 0.38	*p*-value = 0.08
**AGMATINE**	58.51 ± 8.50	59.01 ± 7.39	*p*-value = 0.89
**CADAVERINE**	2.33 ± 0.64	2.35 ± 0.42	*p*-value = 0.93
**ORNITHINE**	2.11 ± 0.42 × 10^3^	1.91 ± 0.49	*p*-value = 0.43
**LISINE**	5.99 ± 0.89 × 10^3^	6.12 ± 0.77	*p*-value = 0.75
**ARGININE**	7.46 ± 0.19 × 10^3^	7.24 ± 0.48	*p*-value = 0.38
**S-ADENOSYL METHIONINE**	202.15 ± 40.26	214.02 ± 35.33	*p*-value = 0.52
**GABA**	43.55 ± 18.42	39.64 ± 13.20	*p*-value = 0.57

## Supplementary Materials

The following supporting information can be downloaded at: https://www.mdpi.com/article/10.3390/diagnostics12092151/s1, Figure S1: Box plots of clinical data (*p* < 0.05) in the three groups (NHL, HL, and HEALTHY), Figure S2: Kruskal-Wallis Box plots of the polyamines in the three groups HEALTHY, NHL and HL. (* *p* < 0.05), Figure S3: Polyamines Box plots in the two groups NHL and HL. The p-value corresponds to the results of the unpaired Student’s *t*-test significant difference for acetyl-spermine (* *p* < 0.008), Figure S4: Box plots of the parameters found to be statistically significant, * *p* < 0.05, in HCV+ and HCV−.

## Abbreviations

NHL	non-Hodgkin’s lymphoma
HLHL	Hodgkin’s lymphoma
HPLC-HRMS	high-performance liquid chromatography
NK	natural killer
B cells	B lymphocytes
T cells	T lymphocytes
WHO	World Health Organization
NLR	neutrophil/lymphocyte ratio ()
dNLR	derived NLR [= neutrophils/(white blood cells − neutrophils ratio)]
PLR	platelet/lymphocyte ratio
MLR	monocyte/lymphocyte ratio
NLR	neutrophil-to-lymphocyte ratio
LMR	lymphocyte-to-monocyte ratio
SIRI	(neutrophil × monocyte)/lymphocyte ratio
AISI	(neutrophil × monocyte × platelet)/lymphocyte ratio
AMC	absolute monocyte count
IPI	International Prognostic Index
DLBCL	diffuse large B-cell lymphoma
MS	mass spectrometry
HPLC-HRMS	high-performance liquid chromatography/high-resolution mass spectrometry
HFBA	Heptafluorobutyric acid
QC	quality control samples
MAD	median absolute deviation
OPLS-DA	orthogonal partial discriminant analysis of the minimum square
PLS-DA	partial least squares discriminant analysis
VIP	variable importance parameter
WBC	white blood cell
RBC	red blood cells
HGB	hemoglobin
PLT	platelet
RDW	red cell distribution
NEUT	neutrophils
LYMPH	lymphocytes
MONO	monocytes
HBV	Hepatitis B Virus
HCV	Hepatitis C Virus
GABA	gamma-aminobutyric acid
SAM	S-adenosylmethionine
