# Supplementary material for: Role of Polyamines as Biomarkers in Lymphoma Patients: A Pilot Study
Source: Diagnostics (Basel). 2022 Sep 4;12(9):2151. doi: 10.3390/diagnostics12092151 (PMC9497571; doi:10.3390/diagnostics12092151)
Supplement: Supplementary file 1 [file diagnostics-12-02151-s001.zip › Supplementary Figures S1-S4.pdf]

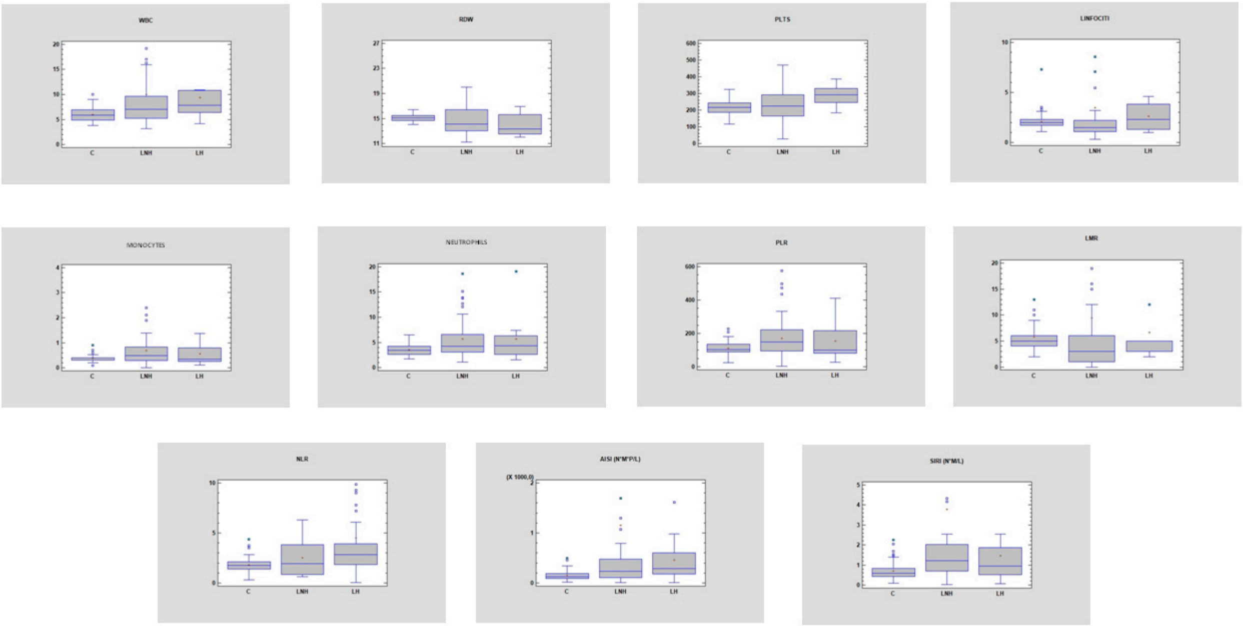

Figure S1. Box plots of clinical data (p<0.05) in the three groups (NHL, HL, and HEALTHY).

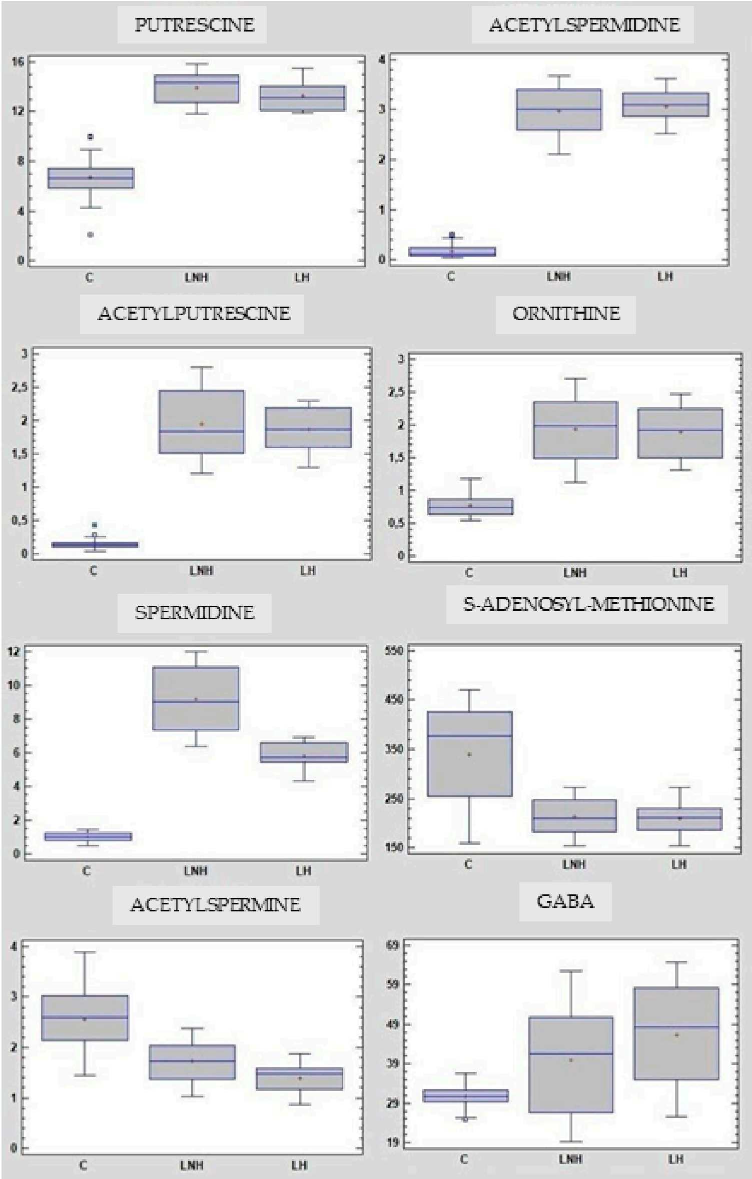

Figure S2. Kruskal-Wallis Box plots of the polyamines in the three groups HEALTHY, NHL and HL. (* p <0.05).

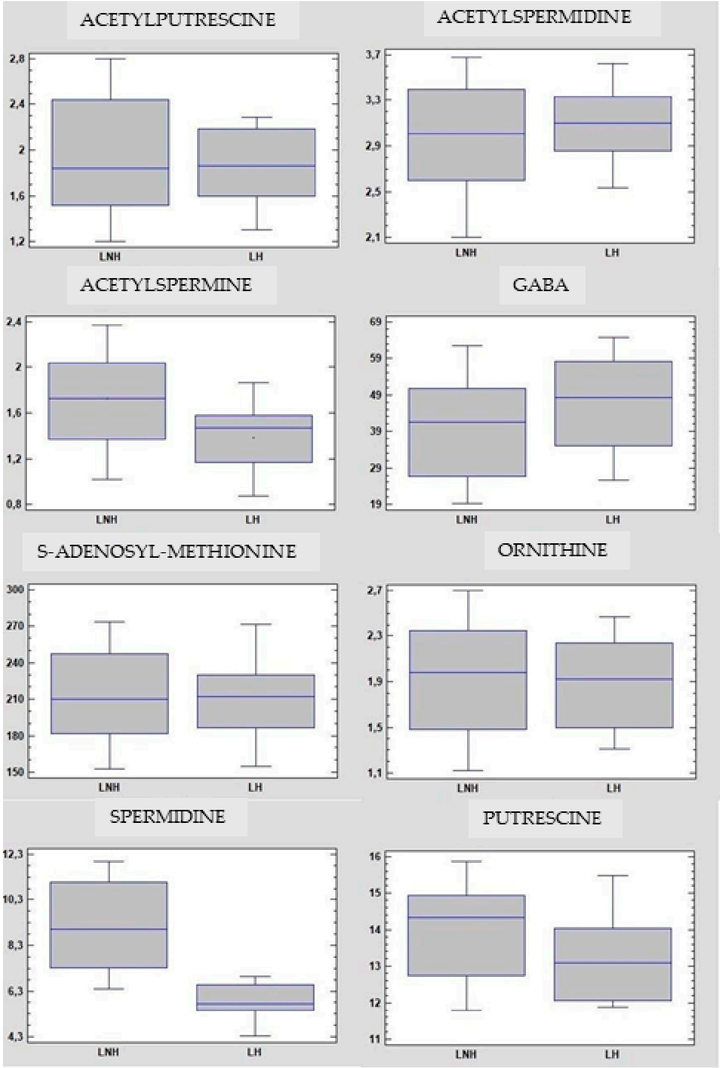

Figure S3. Polyamines Box plots in the two groups NHL and HL. The p-value corresponds to the results of the unpaired Student's t-test significant difference for acetyl-spermine (*p < 0.008).

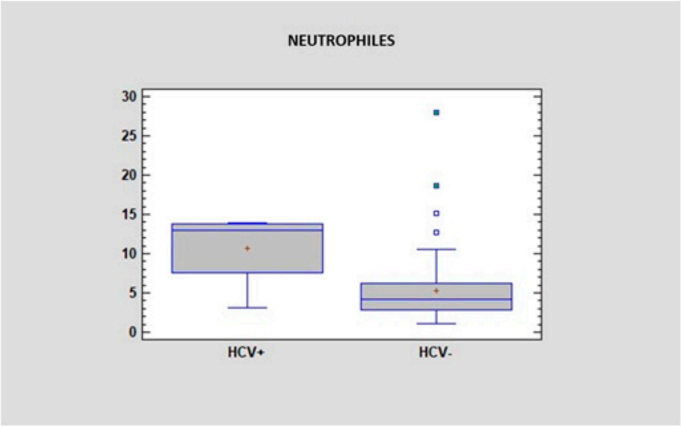

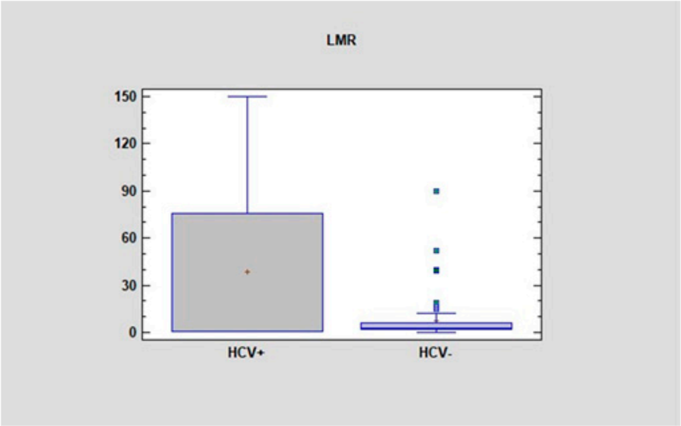

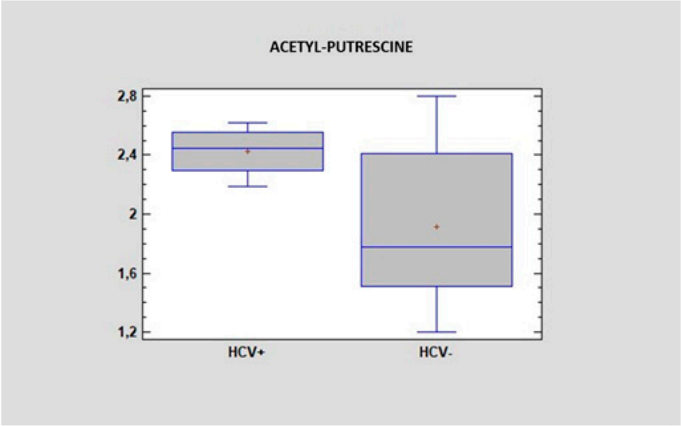

Figure S4. Box plots of the parameters found to be statistically significant, $^*p < 0.05$, in HCV+ and HCV-.
